# Effect of KOB03, a polyherbal medicine, on ovalbumin-induced allergic rhinitis in guinea pigs

**DOI:** 10.1186/1749-8546-7-27

**Published:** 2012-12-20

**Authors:** Hyo Won Jung, Jin Ki Jung, Young Ho Kim, Jong-Seong Kang, Yong-Ki Park

**Affiliations:** 1 Oriental Medicine R&D Center, Dongguk University, Gyeongju, 780-714, Republic of Korea; 2 Department of Herbology, College of Oriental Medicine, Dongguk University, Seokjang-Dong 707, Gyeongju, 780-714, Republic of Korea; 3 College of Pharmacy, Chungnam National University, Daejeon, 305-764, Republic of Korea

## Abstract

**Background:**

KOB03 is a polyherbal medicine that originated from the oriental prescription for the treatment of chronic allergic diseases such as rhinitis and asthma. This study aims to evaluate the effect of KOB03 on ovalbumin (OVA)-induced allergic rhinitis (AR) in guinea pigs.

**Methods:**

Hartley guinea pigs were sensitized to OVA by intraperitoneal injection on days 0, 7, and 14 and challenged with intranasal exposure to OVA three times for 7 days after the last sensitization. KOB03 at doses of 200 and 500 mg/kg were orally administrated to guinea pigs once daily during challenge. The serum levels of histamine, OVA-specific immunoglobulin (Ig) E, eosinophil cationic protein (ECP) and cytokines (TNF-α, IL-4 and IFN-γ) in OVA sensitization/challenge-induced AR guinea pigs were measured. We also observed histological changes in nasal tissues of AR guinea pigs by staining with H&E, Periodic acid-Schiff, and toluidine blue.

**Results:**

The administration of KOB03 at a dose of 500 mg/kg significantly decreased the serum levels of histamine (*P* = 0.001), OVA-specific IgE (*P* = 0.0017), ECP (*P* = 0.008), and TNF-α (*P* = 0.0003) in OVA-sensitized/challenged guinea pigs compared with controls. KOB03 significantly decreased the serum levels of a Th2 cytokine, IL-4 (*P* = 0.017), while significantly increasing the levels of a Th1 cytokine, IFN-γ (*P* = 0.0006) in OVA-sensitized/challenged guinea pigs compared with controls. In addition, KOB03 suppressed the epithelial destruction, goblet cell hyperplasia and eosinophilic infiltration into nasal mucosa associated with AR.

**Conclusion:**

KOB03 may regulate allergic inflammation in AR by inhibiting nasal damage, the release of allergic mediators and modulating the balance of Th1/Th2 cytokines.

## Background

Allergic rhinitis (AR) is an immunoglobulin (Ig) E-mediated inflammatory disease caused by the inflammation of airway mucosa with hypersensitivity resulting from seasonal or perennial responses to specific allergens. It is characterized by a local influx of eosinophils with clinical symptoms including nasal rubbing, sneezing, rhinorrhea, lacrimation, nasal congestion and obstruction [1,2]. AR also triggers systemic inflammation and is associated with various co-morbid conditions such as asthma, nasal polyposis, rhinosinusitis, serous otitis media and sleep disorders [3]. The inflammatory response in the nasal mucosa includes mast cell-mediated allergic responses and a late phase reaction characterized by inflammatory recruitment of secondary effector cells such as eosinophils, basophils, and T cells expressing T-helper 2 (Th2) cytokines including interleukin (IL)-4 (a switch factor for IgE synthesis) and IL-5 (an eosinophil growth factor that promotes allergic inflammation) [4]. In AR, mast cells release inflammatory mediators such as IL-4, IL-5, IL-6, IL-8, IL-12 and TNF-α when activated by IgE-dependent mechanisms [5]. Basophils and eosinophils are also important for allergic inflammation and mainly function in the last phase allergic response. T cells are essential for the regulation and coordination of the adaptive immune response in allergic disease. Th1 T cells release IL-2 and IFN-γ and are involved in delayed type hypersensitivity whereas Th2 T cells release IL-4 and IL-5 and mediate IgE-mediated allergic inflammation [5,6]. Therefore, these cells are considered as major targets for basic and therapeutic research.

In general, the therapeutic principles for AR treatment are to avoid allergens/triggering factors, and symptomatic treatment using different drugs such as antihistamines, decongestants, cromolyn sodium, leukotriene modifiers, nasal glucocorticoids, nasal atropine and allergen immunotherapy (also known as allergy shots), to reduce an individual’s sensitivity to the allergen [5]. Although pharmacotherapy is greatly required, the current treatment for AR is not ideal, as none of these treatments is sufficiently efficacious for nasal obstruction when given as monotherapy [6]. Recently, with a growing global interest in natural drugs, the search for appropriate anti-allergic agents has focused on medicinal plants that are traditionally used in oriental clinics for the treatment of allergic diseases [7]. KOB03 (Table 1) is a polyherbal medicine derived from the oriental prescription for the treatment of hyperhidrosis and allergic diseases such as rhinitis, asthma and itching [8-11].

**Table 1:** Herbal constituents of KOB03

**Herbal name**	**Species**	**Ratio**	**Weight (kg)**
Atractylodis Rhizoma Alba	*Atractylodes japonica Koidz*	2	15.4
Astragali Radix	*Astragalus membranaceus* Bunge	1.5	11.5
Peucedani Japonici Radix	*Saposhnikovia divaricata* Schischkin	1	7.7
Osterici Radix	*Ostericum koreanum* Maximowicz	1	7.7
Scutellariae Radix	*Scutellaria baicalensis* Georgi	1	7.7

This study aims to evaluate the effect of KOB03 on ovalbumin (OVA) sensitization/challenge-induced AR in guinea pigs through regulating the allergic inflammatory response.

## Methods

### Animals

Male Hartley guinea pigs (290–310 g) were purchased from Samtako Co. Ltd. (Gyeonggido, Republic of Korea). The animals were housed under controlled environmental conditions at a temperature of 19–25°C with a relative humidity of 50–60% and 12 h light/dark cycle throughout the study. The care and treatment of animals were in accordance with the guidelines established by the Korean National Institute of Health at the Korean Academy of Medical Sciences for the care and use of laboratory animals and were approved by the Institutional Animal Care and Use Committee of Dongguk University.

### Preparation of KOB03

Herbs used in KOB03 were purchased from the Medicinal Materials Company (Kwangmyungdang Medicinal Herbs, Ulsan, South Korea). All herbs were authenticated by Professor Y.-K. Park, a medical botanist in the Department of Herbology, College of Oriental Medicine, Dongguk University (DUCOM), Republic of Korea. Voucher specimens were deposited in the Herbarium of the DUCOM under registration number 2011-GMP-KOB03-11003. Dried extract of KOB was received from Hanpoong Pharm & Food Co., Ltd. (Jeonju, Korea). Briefly, herbs of KOB03 were mixed according to the ratios in Table 1, minced with a grinder, extracted with 2000 mL of boiling water (95–100°C) for 4 h, and filtered through 50 μm and 1 μm cartridge paper. The filtered extract was concentrated in a rotary evaporator (Hanpoong Pharm & Food Co., Ltd., Jeonju, Korea) at 60°C for 15 h with 700 mmHg, and the concentrated extract was vacuum-dried (yield: 20.0 kg, 40%).

### Preparation of AR guinea pig model

**Figure 1:**
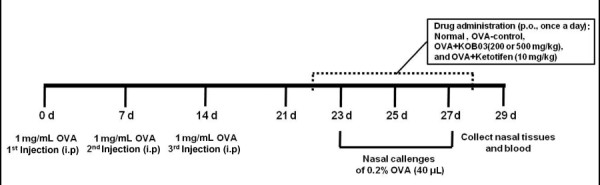
**Study plan for the development of an experimental *in vivo *model of AR.**

The guinea pigs were divided into five groups (*n* = 10 per group) as follows: normal group, OVA-sensitized/challenged group as a control, OVA-sensitized/challenged group with KOB03 at dose of 200 mg/kg or 500 mg/kg, and OVA-sensitized/challenged group with ketotifen at a dose of 10 mg/kg as a reference drug. Guinea pigs were given free access to a standard laboratory diet and water during the experimental period. OVA solution (1 mg/mL in saline) and Al(OH)_3_ (20 mg/mL in saline) were mixed in a 1 to 1 ratio and then mice were sensitized with an intraperitoneal injection on days 0, 7, and 14 (Figure 1). On the seventh day after the last sensitization, the guinea pigs were locally challenged by instilling 0.2% OVA solution (0.04 mL/guinea pig) into the bilateral nasal cavities using a micropipette three times from day 21 to day 28. KOB03 at a dose of 200 or 500 mg/kg body weight or ketotifen at a dose of 10 mg/kg body weight, was orally administered to OVA-sensitized animals once daily for 7 consecutive days during OVA challenge. Normal and OVA-sensitized/challenged animals were given saline alone on the same schedule. Blood samples were taken from each guinea pig by cardiac puncture under isoflurane anesthesia 24 h after the oral administration of KOB03. The administrated doses of KOB03 were calculated from the guidelines for the determination of human equivalent dose (HED) in clinical applications [12]. Serum was prepared and frozen at −70°C prior to analysis. The nasal tissues were removed from the body and histopathological changes were assessed.

### Serological analysis

The concentrations of histamine, OVA-specific IgE, eosinophil cationic protein (ECP) and cytokines such as TNF-α, IL-4, and IFN-γ in the sera of guinea pigs were measured by enzyme-linked immunosorbent assay (ELISA) kits (Wuhan EIAAB Science Co., Ltd., Wuhan, China) or enzyme immunoassay (EIA) kits (Wuhan EIAAB Science Co., Ltd.) according to the manufacturer’s recommendations. The concentration of each substance was calculated from the equations obtained from standard curve plots for the standard solutions in the kits.

### Histological evaluation of nasal mucosal tissues

Guinea pigs were euthanized with a high dose of isoflurane (1–4%) and exsanguinated after final OVA challenge. The head was removed and the lower jaw was discarded. The nasal tissues were separated from the skin, muscle and soft tissue and immersed in freshly prepared 10% neutral buffered formalin for 48 h. The tissue was then rinsed in running tap water and decalcified in 10% nitric acid solution for 5 days. After rinsing in tap water, the tissue was processed for dehydration through graded alcohol and embedded in paraffin. Serial sections (5 μm) were cut at the level of the incisive papillae and first and second palatal ridges and then stained with hematoxylin and eosin (H&E), Periodic Acid Schiff (PAS) for goblet cells and Giemsa for eosinophils. Sections were examined under a light microscope (Leica Microsystems GmbH, Wetzlar, Germany).

### Immunohistochemistry

Specimens were embedded in paraffin, cut into 5-mm-thick sections and floated onto aminoalkylsilane-coated slides (Polysciences, PA, USA). The sections were deparaffinized and rehydrated through graded alcohol washes. After microwave treatment, the sections were treated with 0.3% hydrogen peroxide in methanol for 15 min to inhibit endogenous peroxidase activity of blood cells and with 1% bovine serum albumin (BSA) in 0.05 M phosphate/0.1 M NaCl, pH 7.4 (PBS) containing blocking reagent for another 10 min at room temperature. The sections were incubated with a primary antibody against TNF-α at the appropriate concentration (1:100) in PBS with 1% BSA for 12 h or overnight at 4°C. After thorough washing with PBS containing Triton X-100, each sample was incubated with the secondary antibody (1:100) in PBS with 1% BSA for 3 h at room temperature. After repeated washing, the sections were stained with the ABC kit (Dako, Glostrup, Denmark) that includes DAB for 30 min at room temperature. After washing, the sections were stained with hematoxylin for 1 min (1 drop) at room temperature. After another wash, the sections were dehydrated and mounted with a glass coverslip.

### High-performance liquid chromatography (HPLC) analysis

HPLC analysis for identification of the standard compounds was performed using an HPLC system (Waters, MA, USA) equipped with water 510 pump, waters auto-injector and waters UV/VIS 486 detector. KOB03 powder (1 g) was dissolved in 10 mL distilled water, extracted with ultrasonic waves for 5 min, and filtered through a 0.2-μm PVDF membrane filter. KOB03 (5 mg/mL) or chlorogenic acid (C_16_H_18_O_9_: 354.31; 4 mg), methylvisamminol glucoside (4'-O-beta-D-glucopyranosyl-5-O-methylvisamminol, C_22_H_28_O_10_: 452.46; 5 mg), calycosin glucoside (calycosin-7-O-β-D-glucoside, C_22_H_22_O_10_: 446.1; 1 mg), and baicalin (C_21_H_18_O_11_: 446.37; 21 mg) as standard compounds were dissolved in 1 mL of 100% methanol for pattern analysis using HPLC with a photodiode array detector (Waters, Milford, MA, USA). Chromatographic separation was carried out using an Optimapak C_18_ (4.6 × 250 mm, 5 μm) at room temperature. For analysis of chlorogenic acid, methylvisamminol glucoside, and calycosin glucoside, the injection volume was 10 μL and the following solvent ratios were used for the mobile phase with a flow rate of 1.0 mL/min, where solvent A was 0.5% acetic acid in water and solvent B was 0.5% acetic acid in methanol: 0 min, 25% B; 10 min, 32% B; 20 min, 45% B; 24 min, 48% B; 35 min, 48% B; 40 min, 25% B; and 45 min, 25% B. For analysis of baicalin, the injection volume was 10 μL and the following solvent ratios were used for the mobile phase with a flow rate of 1.0 mL/min, where solvent A was 1% acetic acid in water and solvent B was 1% acetic acid in methanol: 0 min, 30% B; 42 min, 65% B; 44 min, 30% B; and 60 min, 30% B. The detection wavelength (230 nm for chlorogenic acid, methylvisamminol glucoside, and calycosin glucoside or 277 nm for baicalin) was scanned at a flow rate of 1 mL/min at 40°C. The peak analysis and assignment were performed using the standard compounds, which were identified in accordance with their UV spectra and retention time in the HPLC chromatogram.

### Statistical analysis

Data were expressed as means ± standard deviation (SD). Statistical significance was analyzed with one-way analysis of variance (ANOVA), pair-wise and multiple-comparison testing between groups, as well as the Turkey test using GraphPad Prism 5.0 software (GraphPad software, Inc., CA, USA) followed by Dunnet test for individual comparisons. Differences with *P* < 0.05 were considered statistically significant.

## Results

### Allergic mediators

**Figure 2:**
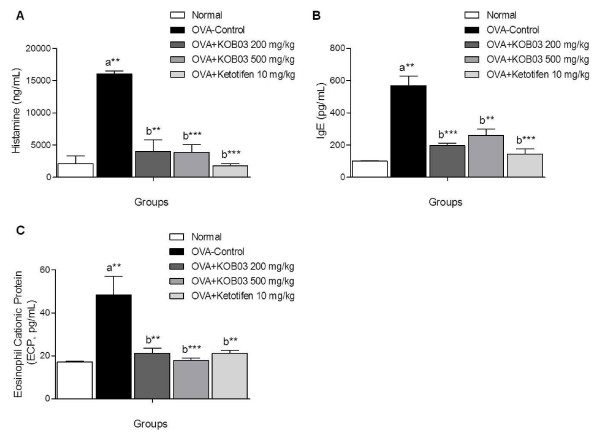
**Effects of KOB03 on the serum levels of allergic mediators in OVA sensitization/challenge-induced AR guinea pigs.** The concentrations of histamine (**A**), OVA-specific IgE (**B**), and eosinophil cationic protein (ECP) (**C**) in the sera of guinea pigs were measured by ELISA. Independent experiments were performed (*n* = 10 per group) and the data shown indicate the mean ± SD. A difference was considered statistically significant when ^**^P < 0.01 and ^***^P < 0.001 *vs.* normal (**a**) or OVA-control (**b**).

To demonstrate the effect of KOB03 on allergic responses *in vivo*, we measured the levels of allergic mediators, histamine, OVA-specific IgE, and ECP in the sera of OVA sensitization/challenge-induced AR guinea pigs. Sensitization/challenge of OVA in guinea pigs induced a significant increase in the production of histamine (Figure 2A; *P* = 0.0044), OVA-specific IgE (Figure 2B; *P* = 0.0019) and ECP (Figure 2C; *P* = 0.0033) compared with the normal group. The administration of KOB03 at doses of 200 and 500 mg/kg significantly decreased the serum levels of histamine (*P* = 0.003 and *P* = 0.001, respectively), OVA-specific IgE (*P* = 0.0005 and *P* = 0.0017, respectively) and ECP (*P* = 0.0016 and *P* = 0.0008, respectively) compared with the control group. The inhibitory effect of KOB03 on the production of allergic mediators was similar to those of the ketotifen-administered group. These data indicate that KOB03 might have anti-allergic activity by suppressing the production of allergic mediators in AR.

### Th1 and Th2 cytokines

**Figure 3:**
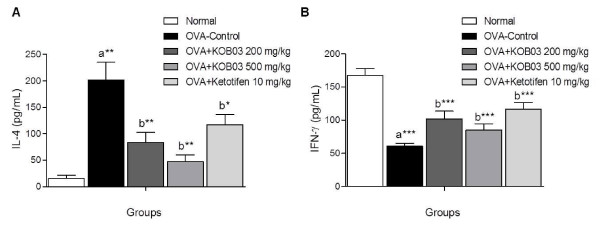
**Effects of KOB03 on the serum levels of cytokines in OVA sensitization/challenge-induced AR guinea pigs.** The concentrations of IL-4 (**A**) and IFN-γ (**B**) in the sera of guinea pigs were measured by ELISA. Independent experiments were performed (*n* = 10 per group), and the data shown indicate the mean ± SD. A difference was considered statistically significant when ^*^P < 0.05 ^**^P < 0.01 and ^***^P < 0.001 *vs.* normal (**a**) or OVA-control (**b**).

To investigate the effect of KOB03 on the cytokine balance *in vivo*, we measured the serum levels of a Th1 cytokine, IFN-γ and a Th2 cytokine, IL-4 by ELISA. Sensitization/challenge of OVA in guinea pigs significantly increased the level of IL-4 (Figure 3A; *P* = 0.005), and significantly decreased the level of IFN-γ (Figure 3B; *P* = 0.0001) compared with the normal group. The administration of KOB03 at doses of 200 and 500 mg/kg significantly decreased the IL-4 levels (*P* = 0.0058 and *P* = 0.0017, respectively), while significantly increasing the IFN-γ levels (*P* = 0.0001 and *P* = 0.0006, respectively) compared with the OVA control group. The regulatory effect of KOB03 on the production of IL-4 and IFN-γ was superior to that of the ketotifen-treated group. These data indicate that KOB03 might control the Th1/Th2 balance in AR.

### Histological changes of nasal tissues

**Figure 4:**
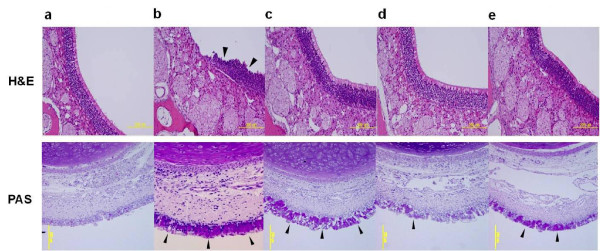
**Effects of KOB03 on histological changes in the nasal mucosa of OVA sensitization/challenge-induced AR guinea pigs.** The histological changes of nasal mucosa were observed by microscope after H&E and PAS staining (magnification × 100). The arrows represent nasal epithelial damage with mucosa edema in the OVA-control group (H&E) and the mucin-positive goblet cells (PAS). **a**, normal; **b**, OVA control; **c**, OVA + KOB03 (200 mg/kg)-administrated group; **d**, OVA + KOB03 (500 mg/kg)-administrated group; and **e**, OVA + ketotifen (10 mg/kg)-administrated group.

**Figure 5:**
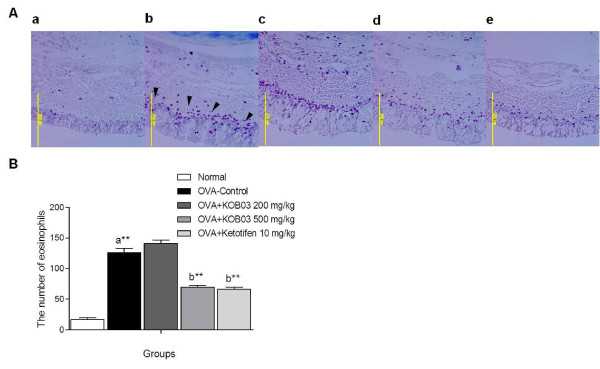
**Effects of KOB03 on the infiltration of eosinophils into the nasal tissues of OVA sensitization/challenge-induced AR guinea pigs. **(**A**) The nasal tissues were stained with Giemsa staining (magnification × 100). The arrows represent Giemsa-positive eosinophils infiltrating the nasal mucosa of guinea pigs. (**B**) The number of eosinophils in the nasal mucosa was counted by observing the sections under a microscope. ^**^P < 0.01 *vs.* normal (**a**) or OVA-control (**b**). **a**, normal; **b**, OVA control; **c**, OVA + KOB03 (200 mg/kg)-administrated group; **d**, OVA + KOB03 (500 mg/kg)-administrated group; and **e**, OVA + ketotifen (10 mg/kg)-administrated group.

Sensitization/challenge of OVA in guinea pigs induced histological changes in the nasal mucosa such as epithelial disruption with mucosa thickening and regional thinning of the epithelium (Figure 4, H&E), the hyperplasia of goblet cells containing mucus (Figure 4) and infiltration of numerous inflammatory cells into the nasal mucosa (Figure 5). The administration of KOB03 at doses of 200 and 500 mg/kg inhibited the nasal destruction and the hyperplasia of mucin-producing goblet cells in OVA-sensitized/challenged guinea pigs. Furthermore, the administration of KOB03 at a dose of 500 mg/kg significantly decreased the numbers of infiltrating eosinophils into the nasal mucosa (*P =* 0.0092) compared with the OVA control group. The inhibitory effect of KOB03 on eosinophil infiltration was similar to that in the ketotifen-treated group. These data indicate that KOB03 might prevent nasal destruction by allergic inflammation during AR.

### TNF-α expression in nasal tissues

**Figure 6:**
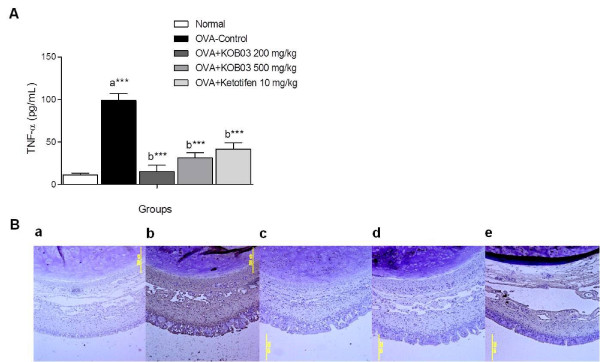
**Effects of KOB03 on TNF-α levels in the serum and in the nasal tissues of OVA sensitization/challenge-induced AR guinea pigs. **(**A**) The concentration of TNF-α in the sera of guinea pigs was measured by ELISA. Independent experiments were performed (*n* = 10 per group), and the data shown indicate the mean ± SD. A difference was considered statistically significant when ^***^P < 0.001 *vs.* normal (**a**) or OVA-control (**b**). (**B**) The expression of TNF-α in the nasal mucosa was observed by immunohistochemistry (magnification × 100). **a**, normal; **b**, OVA control; **c**, OVA + KOB03 (200 mg/kg)-administrated group; **d**, OVA + KOB03 (500 mg/kg)-administrated group; and **e**, OVA + ketotifen (10 mg/kg)-administrated group.

To evaluate the effect of KOB03 on allergic inflammation, we measured the level of TNF-α in the serum of OVA sensitization/challenge-induced AR guinea pigs by ELISA (Figure 6A), and determined its expression level in the nasal mucosa by immunohistochemistry (Figure 6B). Sensitization/challenge of OVA significantly decreased the level of TNF-α in the sera of guinea pigs (Figure 5A; *P* = 0.0007) compared with the normal group. The administration of KOB03 at doses of 200 and 500 mg/kg significantly decreased the level of TNF-α (*P* = 0.0002 and *P* = 0.0003, respectively) in the sera of OVA-sensitized/challenged guinea pigs compared with the OVA-control group. In addition, the administration of KOB03 at doses of 200 and 500 mg/kg remarkably suppressed the expression of TNF-α in the nasal mucosa and were more effective for TNF-α inhibition than ketotifen (Figure 5B).

### HPLC analysis

**Figure 7:**
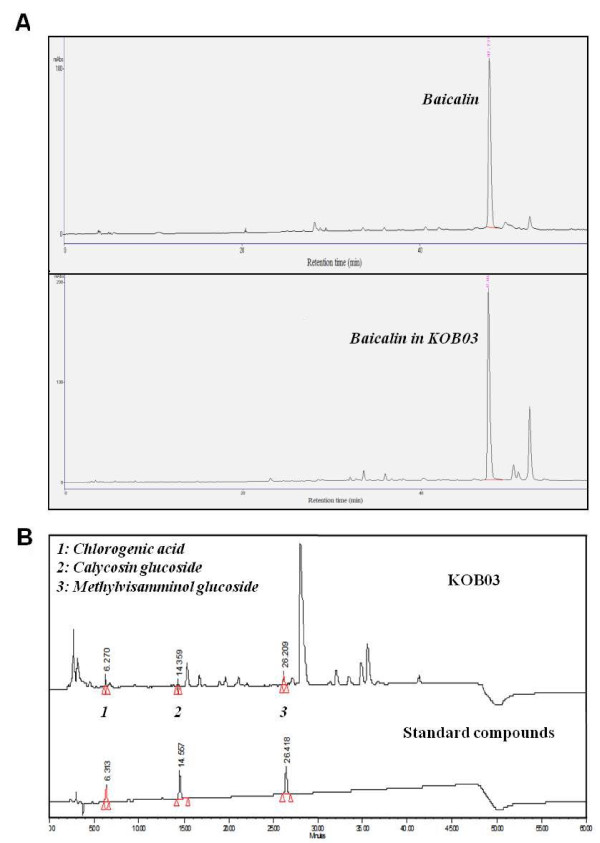
**HPLC pattern analysis of KOB03. **(**A**) HPLC pattern with baicalin. (**B**) HPLC pattern with chlorogenic acid, calycosin glucoside, and methylvisamminol glucoside.

In HPLC analysis of KOB03, the peak assignment was based on the standard compounds, chlorogenic acid, calycosin glucoside, methylvisamminol glucoside, and baicalin in a HPLC chromatogram. Baicalin was identified as the main compound of KOB03 (Figure 7A), and three compounds were also detected in the HPLC analysis (Figure 7B).

## Discussion

Instead of allergen avoidance and environmental control, manipulation of mediator release from airway epithelial cells is the main therapeutic target for managing AR. In addition, treatment strategies have aimed to either reduce the effects of mediators from activated cells in the sensory neural and vascular end organs, or to reduce the tissue accumulation of activated cells that generate inflammatory mediators [5,13]. Although second-generation antihistamines and anticholinergic agents, intranasal corticosteroids and mast cell stabilizers are under development for pharmacotherapy of allergic diseases such as asthma and rhinitis, adverse effects and low clinical efficacy remain [1,2].

Traditional medicines, including complementary, alternative and herbal medicines that improve multi-factorial diseases such as allergic diseases, are considered a good treatment paradigm [14]. KOB03 is a polyherbal medicine derived from herbal prescriptions in oriental clinics, and is a promising candidate for anti-allergic and anti-inflammatory effects in acute and chronic allergic diseases [9,10]. We previously reported that the anti-allergic effect of KOB03 might be due to suppression of mast cell-mediated allergic inflammation [15]. In the present study, we verified that KOB03 had a therapeutic effect on allergic responses in OVA-induced AR in guinea pigs. Among animal models of allergic diseases such as asthma and rhinitis, the guinea pig model of allergic conjunctivitis has proved to be a valid model for the assessment of anti-allergic effects of established and newly developed drugs, especially those targeting mast cell mediators [16].

Sensitization/challenge of OVA in guinea pigs led to increases in serum levels of histamine and antigen-specific IgE and the infiltration of inflammatory cells such as eosinophils in the epithelium and subepithelium of the nasal mucosa [5]. Nasal symptoms of AR are mediated by histamine and inflammatory mediators such as prostaglandins, leukotrienes, trypase and proinflammatory cytokines secreted from activated mast cells, eosinophils and basophils [3,17]. Mast cell-derived mediators collectively induce acute-phase clinical symptoms by enhancing vascular leakage, bronchospasm and activation of nociceptive neurons linked to parasympathetic reflex [18]. In this study, KOB03 markedly inhibited the release of allergic mediators such as histamine and antigen-specific IgE in OVA-sensitized/challenged guinea pigs. These results demonstrated that KOB03 could manage allergic reactions by suppressing mast cell activation in AR similar to the ketotifen-treated group. Ketotifen is a second-generation noncompetitive H1-antihistamine and mast cell stabilizer that helps sufferers of asthma and rhinitis [19].

Eosinophils are inflammatory cells involved in AR [20]. Eosinophils from allergic subjects are primed *in vivo* during allergen challenge, and show increased migratory responses, adhesiveness, degranulation and an altered oxidative mechanism [21,22]. Upon allergen challenge, eosinophils recruited to several organs such as lungs, nose, skin and eyes, and biological fluids, induce the release of oxygen radicals, newly generated mediators such as platelet-activating factor, leukotrienes (LT) C_4_, and ECP [21]. Therefore, eosinophils may be responsible for basic histopathologic changes of target tissues causing vasodilation, mucosal edema, epithelial cell damage, and non-specific hyperreactivity [23]. ECP is a major basic and potentially cytotoxic granule protein secreted by eosinophils. In this study, the administration of KOB03 significantly inhibited the infiltration of eosinophils into the nasal mucosa and decreased the release of ECP from activated eosinophils after sensitization/challenge of OVA in guinea pigs. This suggested that KOB03 protected guinea pigs against allergen-induced allergic immune responses of AR by suppressing the activation of eosinophils.

The pathogenesis of AR involves the allergen-induced proliferation of Th2 lymphocytes secreting IL-3, IL-4, IL-5, IL-9 and IL-13. These cytokines promote IgE production, eosinophil activation and mast cell proliferation [24]. During sensitization, antigen processing by antigen-presenting cells, including dendritic cells, regulates T cell activation, production of specific cytokines and isotype switching. Specific IgE production from B cells mediated by IL-4, is a critical step in the allergic cascade [4,5]. In the present study, the administration of KOB03 significantly inhibited the production of IL-4 in the sera of OVA-sensitized/challenged guinea pigs and inhibited the infiltration of inflammatory cells, eosinophils in the epithelium and subepithelium of the nasal mucosa. This implied that KOB03 might control the imbalance of Th1/Th2 cytokines in AR. Allergic mucosal inflammation characterized by the tissue infiltration of inflammatory cells such as eosinophils, basophils, and neutrophils is associated with the activation of mast cells and T cells.

Proinflammatory cytokines such as TNF-α and IL-1β are potent multifunctional cytokines in the pathogenesis of many inflammatory diseases, including asthma and rhinitis [25]. These cytokines are produced by a variety of cells in the airways and are released by the IgE-dependent activation of mast cells [16]. TNF-α is related to the pathogenesis of these events by upregulating the expression of endothelial cell adhesion molecules, mediating granulocyte chemoattraction, and activating eosinophils, mast cells and T cells [26]. Recent studies [27,28] show that TNF-α may be associated with acquired airway hyperresponsiveness, a pathophysiological hallmark of allergic and inflammatory diseases and can induce the transepithelial migration of neutrophils by IL-8 production and the upregulation of adhesion molecules. In this study, administration of KOB03 significantly decreased the serum level of TNF-α and inhibited its expression in the nasal mucosa of OVA-sensitized/challenged guinea pigs. This suggests that KOB03 might suppress allergic inflammation by the down-regulation of TNF-α expression in AR.

KOB03 is a decoction of five herbs with different pharmacological actions, and the main compound, baicalin, has been identified by HPLC analysis as a standard compound. Baicalin, a flavonoid extracted from various herbal plants, is reported to have antiallergic properties in atopic dermatitis [29], type IV allergic reactions [30], IgE-mediated allergic reactions [31], and anaphylaxis [32]. In addition, three minor compounds, chlorogenic acid as a standard compound of Osterici radix, calycosin glucoside as a standard compound of Astragali radix and methylvisamminol glucoside as a standard compound of Saposhnikoviae Radix were also identified in KOB03 by HPLC analysis. Chlorogenic acid is reported to have antiallergic properties on mast cell-dependent anaphylactic responses in rats [33]. The ability of KOB03 to control the allergic inflammatory response in AR was a result of the synergistic antiallergy and anti-inflammatory effects of its constituents’ components. Although KOB03 has therapeutic potential against AR *in vivo*, further studies should be conducted to identify the active compounds of KOB03 and the mechanism of action of the anti-allergy properties.

## Conclusion

The present study demonstrated that KOB03 has therapeutic potential in AR by inhibiting nasal obstruction and the release of inflammatory mediators such as histamine, allergen-specific IgE, and ECP and modulating the imbalance of Th1/Th2 cytokines in OVA sensitization/challenge-induced AR guinea pigs. These results suggest that KOB03 might be developed as a promising natural medicine for the treatment of allergic rhinitis.

## Abbreviations

AR: Allergic rhinitis; OVA: Ovalbumin; ECP: Eosinophil cationic protein; IgE: Immunoglobulin E.

## Competing interests

The authors declare that they have no competing interests.

## Authors’ contribution

YKP performed botanical evaluation, wrote and revised the manuscript. HWJ conducted histological evaluation statistical analysis. JKJ conducted pharmacological studies. YHK and JSK performed extraction and HPLC pattern analysis. All authors read and approved the final manuscript.
